# Inhibition of the interactions between eosinophil cationic protein and airway epithelial cells by traditional Chinese herbs

**DOI:** 10.1186/1752-0509-4-S2-S8

**Published:** 2010-09-13

**Authors:** Hao-Teng Chang, Louis J Tseng, Ta-Jen Hung, Blacky T Kao, Wei-Yong Lin, Tan-chi Fan, Margaret Dah-Tsyr Chang, Tun-Wen Pai

**Affiliations:** 1 Graduate Institute of Molecular Systems Biomedicine, China Medical University. Taichung, 40402, Taiwan; 2 Graduate Institute of Clinical Medical Science, China Medical University. Taichung, 40402, Taiwan; 3 School of Pharmacology, China Medical University. Taichung, 40402, Taiwan; 4 Institute of Molecular and Cellular Biology & Department of Life Science, National Tsing Hua University, Hsinchu, 30013, Taiwan; 5 Department of Biological Science and Technology, China Medical University. Taichung, 40402, Taiwan; 6 Graduate Institute of Integrated Medicine, China Medical University. Taichung, 40402, Taiwan; 7 Department of Computer Science and Engineering, National Taiwan Ocean University, Keelung, 20224, Taiwan

## Abstract

**Background:**

The eosinophil cationic protein (ECP) is cytotoxic to bacteria, viruses, parasites and mammalian cells. Cells are damaged *via* processes of pore formation, permeability alteration and membrane leaking. Some clinical studies indicate that ECP gathers in the bronchial tract of asthma sufferers, damages bronchial and airway epithelial cells, and leads to in breathing tract inflammation; therefore, prevention of the cytotoxicity caused by ECP may serve as an approach to treat airway inflammation. To achieve the purpose, reduction of the ECP-cell interactions is rational. In this work, the Chinese herbal combinative network was generated to predict and identify the functional herbs from the pools of prescriptions. It was useful to select the node herbs and to demonstrate the relative binding ability between ECP and Beas-2B cells with or withour herb treatments.

**Results:**

Eighty three Chinese herbs and prescriptions were tested and five effective herbs and six prescription candidates were selected. On the basis of effective single-herbal drugs and prescriptions, a combinative network was generated. We found that a single herb, Gan-cao, served as a node connecting five prescriptions. In addition, Sheng-di-huang, Dang-guei and Mu-tong also appeared in five, four and three kinds of prescriptions, respectively. The extracts of these three herbs indeed effectively inhibited the interactions between ECP and Beas-2B cells. According to the Chinese herbal combinative network, eight of the effective herbal extracts showed inhibitory effects for ECP internalizing into Beas-2B cells. The major components of Gang-cao and Sheng-di-huang, glycyrrhizic acid and verbascose, respectively, reduced the binding affinity between ECP and cells effectively.

**Conclusions:**

Since these Chinese herbs reduced the binding affinity between ECP and cells and inhibited subsequent ECP entrance into cells, they were potential for mitigating the airway inflammation symptoms. Additionally, we mentioned a new concept to study the Chinese herbs using combinative network in the field of systems biology. The functional single herbs could be identified from the set of prescriptions.

## Introduction

Eosinophil cationic protein (ECP) is a human protein secreted from activated eosinophils and released into biological fluid under inflammatory conditions [1]. It belongs to the ribonuclease A (RNaseA) superfamily which is composed of structure- and sequence-related members that possess hydrolytic activities towards polymeric ribonucleic acids (RNA) for regulating gene expression at the mRNA level [2]. Nine human proteins with significant sequence similarities to bovine pancreatic RNaseA, the first isolated RNase, are grouped into the human RNaseA superfamily and are named from RNase1 to RNase9 [3-5]. Within this protein superfamily are two categories: one possesses high ribonucleolytic activity, including RNase1, RNase2, RNase4, RNase6, and RNase8, and the other is cytotoxic towards bacteria, parasites, or mammalian cells, such as RNase3 (ECP), RNase7 and RNase9 [4,6-8]. Structurally, ECP folds topology containing 3 α-helices and 5 β-strands [9]. It is a highly positively-charged protein due to its high Arg content (19 Args, pI=10.8), which promotes interactions between ECP and the molecules with negative charges on the cell surface, such as proteoglycan, heparin sulfate and lipid molecules [10,11]. Through the interactions with organism surface, ECP translocates into cells and causes cell damage [12]. In addition to cytotoxicty, ECP also displays antimicrobial activities against bacteria, single strand RNA viruses, and helminth parasites and mammalian cells [13-15]. Recent reports have mentioned that the *N*-terminus of ECP is bactericidal and disrupts lipid membrane of the target cells [16].

Some hypothesized mechanisms of ECP-triggered cell damage suggested that ECP destabilized cell membrane *via* processes of pore formation, permeability alterations and membrane leaking [11]. ECP contributed to tissue damage with regards to bronchial asthma [17] or the intestinal mucosa in Crohn’s disease [18]. In clinical diagnosis, it has been used as a biomarker for determination of the severity of airway inflammatory diseases, such as asthma [17]. The ECP level in serum also increased during a pathogen infection, such as toxocara [19] and cystic hydatidosis [20]. During airway inflammation, ECP was a common feature to indicate bronchial epithelial cell damage [21]. It was also reported to damage Beas-2B cells through the apoptosis mechanisms of caspase 8 [22]. We have reported that the carboxypeptidase E (CPE) and heparan sulfate proteoglycans served as receptor-like molecules for ECP binding to cell membrane and translocating into neuroendocrine cells [23] and bronchial epithelial cells [12], respectively. In 2008, the heparin binding residues in ECP were also identified and characterized [24]. 

Since cell damage caused by ECP is one of the significant features of airway inflammation, it is of great interest in discovering practical substance to neutralize the cytotoxicity of ECP. As is known, the cytotoxicity of ECP can be blocked by specific antibodies and heparin *in vitro *[20,24], the aim of this study is to employ combinative network analysis to discover a potent substance which has been widely used and is effective for inhibiting the cytotoxicity of ECP. This methodology assisted in identifying the potent effective components from prescriptions or herbs; therefore, the herbal variety may be decreased and the formulation of drugs can be simplified. Similar to the strategy, the combinative therapy is used. The combinative therapy can present synergistic effect to strengthen the drug efficacy but using less drug dosage. This therapy concept is similar to the treatment of traditional Chinese medicine, in which prescriptions are formulated from single herbs. In addition, the systems biology approaches for studying combinative therapy have been established in the levels of genome wide transcriptome and proteome on cancer researches [25-27]. 

For a long time, Chinese people have used many herbs and prescriptions to treat asthma and airway inflammation. In Chinese medical science, several prescriptions have been available for supportive care against the diseases caused by the patients’ own immune system. Asthma sufferers can be treated by supporting approaches to regulate the whole body’s immunity, but these strategies are too slow to respond effectively in the emergent events. In this report, we characterized some of the Chinese herbs which inhibited interactions between ECP and the airway epithelial cell line so that the cytotoxicity of ECP might be reduced or eliminated. According to the wet lab experimental data, we generated Chinese herbal combinative network to identify the node herbs which were the components of effective prescriptions. In total 83 kinds of Chinese prescriptions and herbs recommended by Chinese medical doctors were screened using ELISA method. The decrease in binding affinity between ECP and bronchial epithelial cells could be quantified. Moreover, the effective Chinese herbs also inhibited the ECP translocating into Beas-2B cells at very low concentration. Most of the major compounds of effective Chinese herbs also showed inhibitory effects on reducing the binding affinity between ECP and cells. They may serve as the potent drugs for the development of anti-asthma or anti-inflammation treatments.    

## Results and discussions

### The pre-screening of Chinese herbs

In the first step, thousands of Chinese herbs and prescriptions were prescreened by Chinese medical doctors. After prescreening, two herbal sets containing 90 and 83 kinds of herbs or prescriptions were selected and purchased from Sun Ten Pharmaceutical Co. Ltd. and Chuang Song-Zong Pharmaceutical Co. Ltd., separately. Because of the manufacture process, the single-herbal drugs and prescriptions from Chuang Song-Zong Company were boiled, cooled and concentrated to viscous formulation; however, the drugs from Sun Ten Company appeared clear. After ELISA analysis, the drugs from Sun Ten Company showed less effect on inhibiting the ECP-cell interactions, possibly due to the difference between the two manufacture processes. As a result, the herbs produced by Chuang Song-Zong Company were screened to select the effective candidates for reducing the binding affinity of ECP and cells. The English and Chinese names of herbs used in this study was listed in Additional file 1.

### Eleven herbs and prescriptions reduced 90% of the ECP-cell affinity

**Figure 1:**
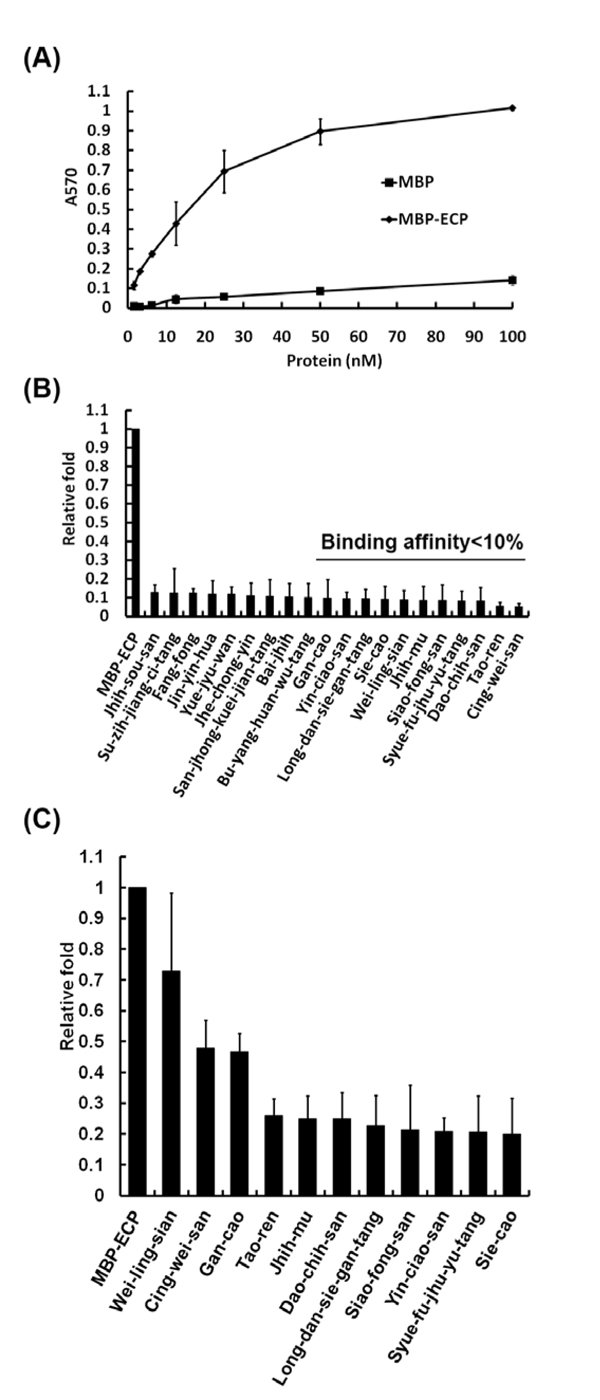
**Herbal extract effects on ECP-cell binding affinity.** The comparison of MBP and MBP-ECP interactions with Beas-2B cells (A). Partial results of MBP-ECP-cell interaction in the presence of 0.1% (B) and 0.01% (C) herbs. The line shown in (B) means the relative fold less than 10%.

**Figure 2:**
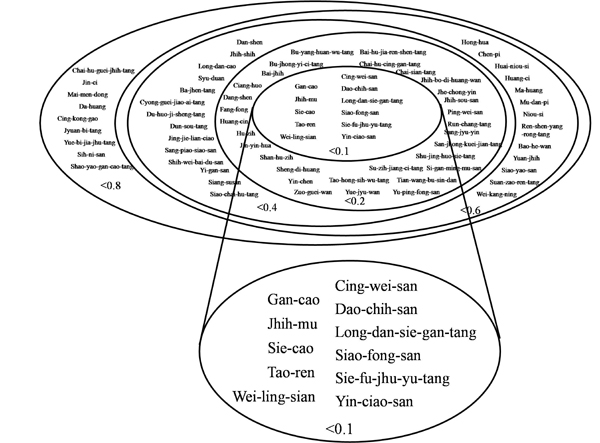
**Classification of Chinese herbs inhibiting the interactions between ECP and Beas-2B cells.** The inhibitory effect is classified into 5 groups from less than 10% to 80% as indicated. The most effective drugs containing 5 herbs and 6 prescriptions were zoomed in as illustrated.

The cell-based ELISA platform is employed to screen for drugs that can reduce or inhibit the interactions between ECP and Beas-2B cells. It is expected that the cytotoxicity of ECP can be inhibited *via* blockage of the ECP-cell interactions. ECP was tagged with MBP for antibody probing in the ELISA process. As shown in **Fig. **1A, MBP-ECP, compared with MBP, significantly bound to cells in a concentration-dependent way. Prior to ELISA, 83 herbs or prescriptions were individually mixed with 100 nM MBP-ECP recombinant proteins to a final concentration of 0.1% (w/v) in RPMI-1640 serum-free media. The relative binding affinity normalized by MBP-ECP-cell binding strength was measured and calculated (Most of data is not shown and partial data is in **Fig. **1B). As shown in **Fig. **2, the herbs and prescriptions were classified into 5 groups according to the relative reductive folds of ECP-cell binding affinity. Five herbs and 6 prescriptions reduced 90% of the affinity in ECP-cell interaction (**Fig. **1B); however, **Fig. **1B also indicated that although 11 herbs inhibited ECP-cell interaction most effectively, some other herbs also reduced interactions significantly. In order to verify the effectiveness of drugs, the central 11 herbs or prescriptions listed in **Fig. **2 were analyzed afterwards. As they were diluted to a final concentration of 0.01%, most of these drugs, excluding  Wei-ling-sian, still reduced ECP-cell interaction significantly (**Fig. **1C). 

### Gan-cao, Sheng-si-huang, Dan-guei and Mu-tong were potential for inhibiting the ECP-cell interactions

**Figure 3:**
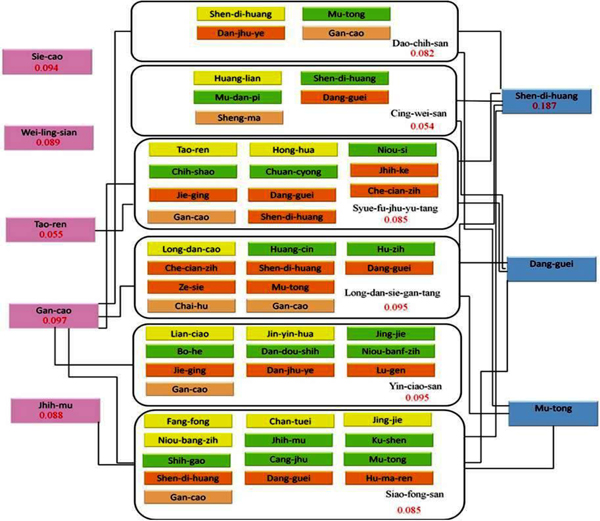
**The network combined with herbs and prescriptions.** The herbal prescriptions are gathered and boxed. Each herb is separately shown in yellow, green, dark orange and light orange boxes, which means Jun, Chen, Zuo and Shi, respectively. The consensus herbs in prescriptions are linked and connected as nodes. The relative fold of the inhibition of ECP-cell interactions is shown in red numerals.

**Figure 4:**
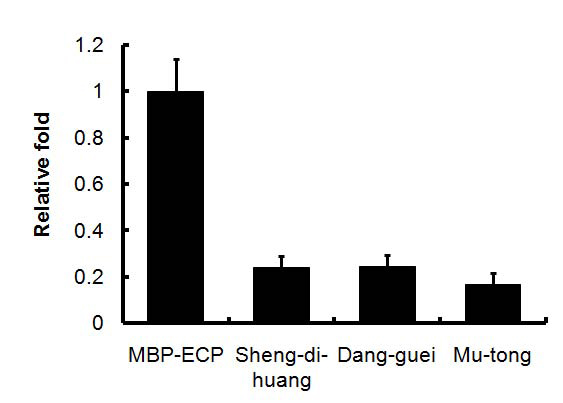
**The binding affinity between ECP and cells is decreased by adding Sheng-di-hunag, Dang-guei and Mu-tong.** Compared with MBP-ECP control, the decrease of binding affinity is significant (*P*<0.001). Each experiment is triplicated.

The 83 drugs were divided into two groups: single herbs and prescriptions. Cross comparison provided useful information to verify the effective herb for further investigation. According to the ELISA results, Gan-cao and Sheng-di-huang appeared in 5, Dan-guei in 4 and Mu-tong in 3 of the 6 prescriptions (**Fig. **3 ), among which Gan-cao displayed the most potential. In the combinative network, Gan-cao served as a node linked to prescriptions, Dao-chih-san, Long-dan-sie-gan-tang, Siao-fong-san, Sie-fu-jhu-yu-tang and Yin-ciao-san, and it could reduce 90% of ECP-cell affinity at a low concentration of 0.1% (w/v). Although in the Chinese medicine concept, Gan-cao is “Shi”, which plays a role in assisting the sound effects of “Zuo” herbs, it may play a novel role in mitigating the symptoms of inflammation. Besides Gan-cao, another node, Sheng-di-huang, also appeared in 5 prescriptions and reduced approximately 80% of the ECP-cell binding affinity at a concentration of 0.1% (w/v) (**Fig. **4 ). Consequently, it may serve as a new candidate for inflammation treatment. Dan-guei and Mu-tong were two nodes in the combinative herbal network. However, they were not selected by Chinese medical doctors in the pre-screening processes. After the cell-based ELISA was performed, Dan-guei and Mu-tong at a concentration of 0.1% (w/v) exactly decreased the affinity between ECP and Beas-2B cells to 24.4% and 16.8%, respectively **(Fig. **4). According to the results of Dan-guei and Mu-tong, it is demonstrated that our approaches to analyzing the combinative network of the herbs can identify effective drugs from the complex prescription formula.

### The inhibition of ECP translocating into Beas-2B cells

**Figure 5:**
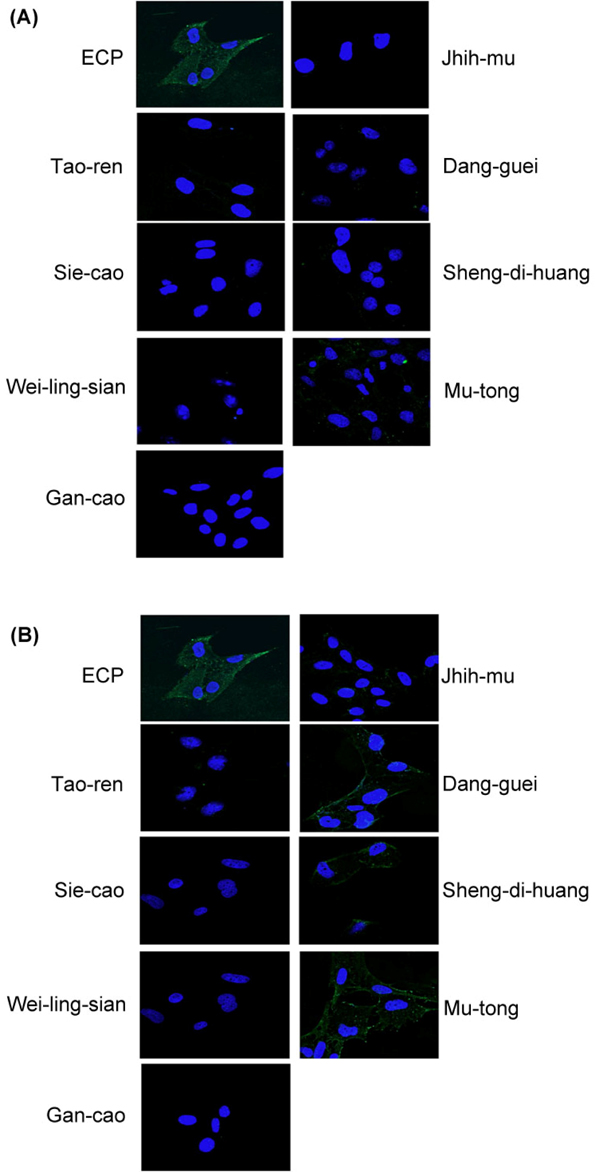
**MBP-ECP tanslocation is inhibited by the Chinese herbs.** The recombinant MBP-ECP (200 nM) is pre-incubated with Chinese herbs at a concentration of 0.1% (A) and 0.01% (B). The nucleus is in blue and MBP-ECP in green.

According to the cell-based ELISA experiments and the analysis of combinative network, 8 herbs, Tao-ren, Sie-cao, Wei-ling-sian, Gan-cao, Jhih-mu, Dang-guei, Sheng-di-huang, Mu-tong, reduced the binding affinity between ECP and cells. Therefore, they should inhibit ECP translocating into Beas-2B cells. MBP-ECP was mixed with herbal extracts at a concentration of 0.1% **(Fig. **5A) or 0.01% **(Fig. **5B) and incubated with the Beas-2B cells for 1 hr. Employing the immunofluorescent technique, MBP-ECP was traced using anti-MBP antibodies and shown through green fluorescence. In the treatment with 0.1% herbal extracts, all of the 8 herbal extracts inhibited the translocation of ECP into Beas-2B cells. Sheng-di-huang, Dang-guei and Mu-tong under the treatment at a concentration of 0.01% showed less inhibitory effect on ECP translocation. It is proposed that different herbs inhibited ECP translocation under different concentrations with various components. As a result, the major components of some effective Chinese herbs were selected for further investigation. 

### The major components of herbs inhibited the interaction of ECP and Beas-2B cells

**Figure 6:**
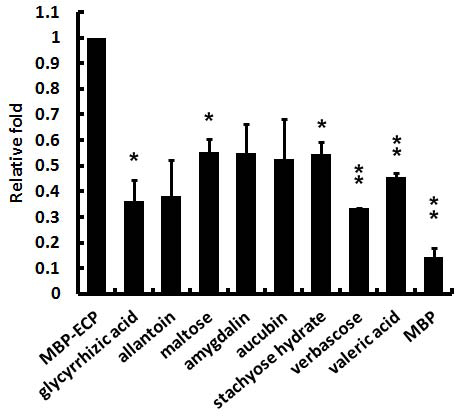
**Binding affinity between ECP and cells is decreased by adding the major components of the Chinese herbs.** Compared with MBP-ECP, the binding affinity of verbascose and valeric acid are decreased significantly (** *P*<0.02) and stachyose hydrate, maltose and glycyrrhizic acid are minor significant (* *P*<0.06). Each experiment is duplicated.

Chinese herbs have been studied for a long time and some major components can be purchased from commercial chemical companies. Listed in **Table **1 , most of the major components are carbohydrate, such as sugars and acids. Previous studies have shown that ECP interacts with proteoglycan, heparin sulfate, and condrotin sulfate [12,24]. It meant the major components derived from Chinese herbs might inhibit the ECP-cell interaction effectively. **Fig. **6 shows at a concentration of 50 nM, the major components of herbs decreased 40%-70% binding affinity between ECP and cells. Among them, the major components of Gang-cao and Sheng-di-huang, glycyrrhizic acid and verbascose, respectively, showed the most efficient inhibition towards ECP-cell interactions. Because the decrease of binding affinity between ECP and cells was similarly ranging from 0.05-1.6 μM (**Additional file **2 ), other components of herbs, which we did not characterize, might play potential roles in interacting with ECP and preventing ECP binding with cells.

**Table 1:** Major components of Chinese herbs

Chinese Herbs	Pharmaceutical species	Major components
Gan-cao	*Glycyrrhizae Radix*	Glycyrrhizic acid
Tao-ren	*Prunus persica Batsch*	Allantoin
Tao-ren	*Prunus persica Batsch*	Maltose
Tao-ren	*Prunus persica Batsch*	Amygdalin
Sheng-di-huang	*Rehmanniae Radix et Rhizoma*	Aucubin
Sheng-di-huang	*Rehmanniae Radix et Rhizoma*	Stachyose
Sheng-di-huang	*Rehmanniae Radix et Rhizoma*	Verbascose
Sie-cao	*Valeriana officinalis*	Valeric acid

In Chinese prescription, the herbs are separated into 4 parts, Jun, Chen, Zuo and Shi; Jun is the major drug entity to target a disease; Chen either supports Jun or reduces the side effects of Jun; Zuo makes Jun and Chen stronger; it reduces the adverse effects or eliminates the toxicity of Jun and Chen; Shi harmonizes the total herbs or guides the herbs to the target effectively [28]. Although Gang-cao plays the role as Shi, and Sheng-di-huang plays as Jun, Chen, and Zuo in some prescriptions, they may have the potential to be developed as anti-airway inflammation drugs to prevent airway damage. After the major effective compounds are identified, we may adapt the concept of Jun, Chen, Zuo and Shi to develop combinative drugs or formulations to make drugs more efficient, effective and safe. 

## Conclusions

It is important to develop an approach for analyzing the complex components of tranditional herbs or prescriptions. This paper provides a methodological concept to select the potential single herbs from several effective prescriptions. Employing the same strategy and combinative network, we may select the potential compounds from a effective herb. Here, we identified the major components of Gan-cao and Sheng-di-huang which inhibited the interactions of ECP and Beas-2B cells. These compounds might serve as potential drugs to rescue airway epithelial cells damaged by ECP. 

## Materials and methods

### The flowchat 

**Figure 7:**
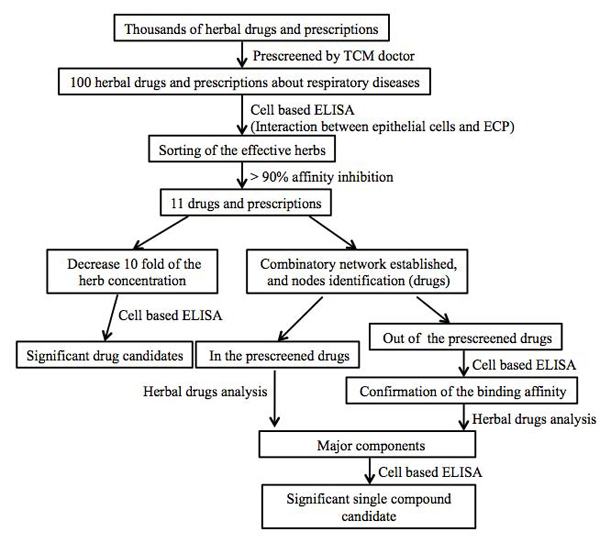
The flowchat of this study.

Drug screening approaches provided clear information to validate the efficacy of each drug candidate. However, it is costly and time-consuming. Our methodology used in this study combined with wet and dry lab methods to screen the potential drugs which could inhibit the interaction between ECP and bronchial cells. Drug prescreening by tranditional medical doctor cut down the number of drug candidates to 173. Cell-based ELISA systems provided efficient platform to screen the effective herbs. The combinative network analysis presented the node herbs which might play an important role in inhibiting the ECP-cell binding. At last, the major compounds of effective herbs were selected to detect the inhibitive activity towards ECP-cell interaction (**Fig. **7 ).

### Antibodies and reagents

Antibodies were obtained from the following sources: mouse anti-MBP from Santa Cruz Biotechnology (USA); Goat anti-mouse HRP-from Jackson (USA); Goat anti-mouse FITC-conjugated from Abcam (USA); Hoechst 33342 from Molecular Probes (USA); RPMI 1640 medium from Sigma-Aldrich (USA); fetal bovine serum (FBS) from Gibco/Invitrogen (USA) and tetramethylbenzidine (TMB) from KPL (USA).

### Cells and cell culture

Beas-2B, a human bronchial epithelial cell line (ATCC CRL-9609), was cultured in RPMI-1640 medium supplemented with 10% heat-inactivated fetal bovine serum at 37°C in an incubator containing 5% CO_2_ and 95% air.

### Purification of recombinant proteins 

The pMAL-c2X-ECP was transformed into *E. coli* BL 21(DE3) pLysS (Novagen, USA) for protein expression. Twenty milliliters of the culture grown overnight were inoculated into 500 ml of LB media containing 100 µg/ml carbenicillin (Sigma-Aldrich, USA) and 50 µg/ml chloramlphenicol (Sigma-Aldrich, USA), followed by incubation at 37°C for 6 hr till the OD_600_ reacβ-D-1-thiogalactopyranoside (IPTG) (Ameresco, German) was added to a final concentration of 1 mM, and the bacteria were harvested after a 4-hr induction at 30°C. Recombinant MBP-ECP was fractioned in the soluble portion of bacterial lysate. MBP-ECP was purified using amylose affinity chromatography (NEB, UK) and switched to PBS buffer. To remove the impurity, heparin affinity chromatography was utilized. Then, MBP-ECP was eluted using 100 mM phosphate buffer (pH 7.4) containing 1 M NaCl. The purified MBP-ECP was concentrated and the buffer was changed to PBS by Amicon Ultra-15. The protein concentration of MBP-ECP was determined by BCA assay kit (Pierce, USA) and the MBP was also purified and served as the negative control for cell-based ELISA.

### Preparation of Chinese herbs 

The extracts of Chinese herbs were viscous, so they were diluted into distilled water to a final concentration of 10% (w/v). Afterwards, the mixture was centrifuged at 12,000 *g* at 4°C for 20 min, and the supernatant was transferred to a new tube and stored at -20°C. In the cytotoxcity assay, the herbs were filtered by 0.22 μm.

### Cell-based ELISA

The binding affinity between MBP-ECP and Beas-2B cells in the presence of various Chinese herbs was measured using cell-based ELISA. One microliter of the processed herbs was diluted into 49 μl serum-free RPMI-1640 medium containing 100 nM MBP-ECP. The Beas-2B cells with 70% confluent monolayer in each well of a 96-well plate (20,000 cells/well) were washed with PBS and blocked with 2% BSA/PBS at 4°C for 1 hr. Next, the cells were incubated in 50 μl serum-free media and mixed with 50 μl herb/MBP-ECP mixture at 4°C for 1 hr. After being washed with PBS, fixed with 2% PFA/PBS, quenched with 50 mM NH_4_Cl and reblocked with 2% BSA/PBS, the MBP-ECP was probed by mouse anti-MBP mAb at 25°C for 1 hr and anti-mouse HRP-conjugated secondary antibody was used to probe the anti-MBP mAb. One hundred microliters of TMB were added into each well prior to incubation for 10 min in the dark. After terminating the reaction by adding 100 μl 2N HCl, the absorbance for 450 nm was measured using ELSA reader (Biotek, Australia). The *A_450_* value indicating the interaction between MBP-ECP and cells was set as 100% and the relative fold of ECP-cell binding reduction was calculated using Microsoft Office Excel 2007.

### Drug treatment

Beas-2B cells were seeded on coverslips into 24-well culture plates (100,000/well) in RPMI 1640 medium containing 10% FBS and incubated for 2 days to form a monolayer at the bottom of plates. In addition, cells were incubated at 37°C for 1 day in serum-free RPMI 1640 medium for serum starvation. Eight herbal extracts, Tao-ren, Sie-cao, Wei-ling-sian, Gan-cao, Jhih-mu, Dang-guei, Sheng-di-huang, Mu-tong, at concentrations of 0.1% or 0.01% (w/v) were mixed with 200 nM MBP-ECP for 30 min at room temperature and treated with Beas-2B cells at 37°C for 1 hr. The major component of Chinese herbs listed in **Table **1 was also tested at concentrations of 0.05-1.6 μM.

### Generation of combinative network

A combinative network was generated according to the ELISA screening results. Each herb of the prescriptions was listed and boxed. The network was generated using CellDesigner 4.0.1 [29] and modified using Microsoft Office Powerpoint 2007.

### Immuno-fluorescence staining and confocal microscopy

After drug treatment, cells were extensively washed with PBS, fixed with 2% paraformaldehyde (PFA) in PBS for 15 min, quenched with 50 mM NH_4_Cl in PBS for 10 min, and then permeabilized with 0.5% (v/v) Triton-X-100 for 5 min at room temperature in turn. The cells were blocked with 2% BSA in PBS for 30 min at room temperature, and labeled with primary antibodies diluted in 0.5% BSA in PBS for 1 hr at 25°C, followed by washing two times with 0.05% (v/v) Triton-X-100 and one time with PBS, and then incubated with secondary antibodies diluted in 0.5% BSA in PBS for 1 h at 25°C. Nuclei were stained with Hoechst 33342 during the last 5 min of incubation. Cells were then washed two times with 0.05% Triton-X-100 and one time with PBS, and coverslips were mounted by ProLong^®^ Gold antifade reagent (Invetrogen). Confocal microscopy was performed employing an Axiovert (Zeiss) confocal microscope using the 63 × Plan-NEOFLUAR oil immersion objective. 

## Abbreviations  

ECP: eosinophil cationic protein; MBP: maltose-binding protein; RNase: ribonuclease; ELISA: enzyme-linked immunosorbent assay

## Competing interests

The authors declare that they have no competing interests.

## Authors' contributions

HTC conceived this study and drafted the manuscript. LJT conducted the cell-based ELISA and drug screening. TJH finished the confocal microscope studies. BTK participated in the drug screening. WYL helped to pre-screen the Chinese herbs in the initial design. TCF and MDTC helped the establishment of the cell-based ELISA. TWP participated in the network generation.

## Supplementary Material

Additional file 1 The English and Chinese names of herbs used in this study. Click here for file

Additional file 2 Relative binding affinity between ECP and cells is decreased by adding different concentrations of the major components of the Chinese herbs. Each experiment is duplicated. Click here for file
